# Fracture nonunion

**DOI:** 10.11604/pamj.2021.40.93.28184

**Published:** 2021-10-13

**Authors:** Pragadeesh Palaniappan, Krishna Prasanth Baalann

**Affiliations:** 1 Department of Community Medicine, Sree Balaji Medical College and Hospital Biher, Chennai, India

**Keywords:** Fracture, nonunion, malunion, forearm

## Image in medicine

A fracture that lasts for a minimum of nine months without signs of healing for three months is the standard description of nonunion. Plethora of literatures highlights the determinants that increase the risk of nonunion as infections, older age, diabetes, anti-inflammatory drugs, use of tobacco/nicotine, open/compound fracture, low vitamin-D, hypothyroidism, poor nutrition, severe anaemia, etc. Apart from these, there are hidden factors influencing the occurrence of nonunion. This article emphasises on such hidden factors. This case is a 70 year old man who came with complaints of cold and cough. Incidentally, the abnormal appearance and mobility in the forearm was noticed (A). Further history revealed that, the patient had a past history of road traffic accident 10 years back, followed by which he took native treatment from traditional bone setters. He´s a known case of diabetes, hypertension. On examination, patient had painless abnormal movement in the upper 1/3rd of the forearm. The distal part of forearm was moving horizontally, vertically and rotationally. Radiological image shows complete fracture of both bones in forearm (B). In fracture treatment, patient compliance is very crucial. In this case, the patient was initially treated by a local traditional bone setter. The patient is unaware of the treatment options available in modern medicine for such fractures. The patient´s poor socioeconomic status, nutritional status, smoking habit (30 years) also played a vital role in poor bone healing. In developing countries like India, this case scenario throws light on the huge lacunae in health education and perception regarding fracture management among people in the rural areas.

**Figure 1:**
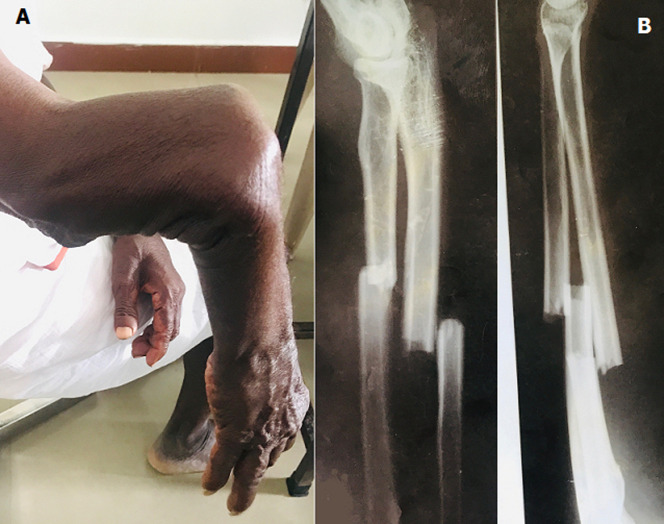
A) forearm with fracture nonunion; B) X-ray showing fracture both bones in forearm

